# A middle - late Eocene neoselachian assemblage from nearshore marine deposits, Mahajanga Basin, northwestern Madagascar

**DOI:** 10.1371/journal.pone.0211789

**Published:** 2019-02-27

**Authors:** Karen E. Samonds, Tsiory H. Andrianavalona, Lane A. Wallett, Iyad S. Zalmout, David J. Ward

**Affiliations:** 1 Department of Biological Sciences, Northern Illinois, University, DeKalb, Illinois United States of America; 2 Domaine des Sciences et Technologies, Mention Bassins Sédimentaires Evolution Conservation, Université d’Antananarivo, Antananarivo, Madagascar; 3 Department of Integrative Physiology and Neuroscience, College of Veterinary Medicine, Washington State University, Pullman, Washington, United States of America; 4 Department of Paleontology, Saudi Geological Survey, Jeddah, Saudi Arabia; 5 Department of Earth Sciences, The Natural History Museum, London, United Kingdom; Royal Belgian Institute of Natural Sciences, BELGIUM

## Abstract

We report here the first neoselachian fossil fauna from Eocene nearshore marine deposits of the Mahajanga Basin, northwestern Madagascar. The fauna includes seven species of shark: *Nebrius blankenhorni*, *Brachycarcharias koerti*, *Galeocerdo eaglesomei*, two species of *Carcharhinus* (one of which is described as a new species), *Physogaleus*, *Rhizoprionodon and Sphyrna*. Three species of rays were also recovered: *Pristis*, *Myliobatis* and an undetermined dasyatid ray. This fauna represents the first Cenozoic neoselachian fossil record from the Eocene of Madagascar and broadens our understanding of their evolutionary and biogeographic history in the southern hemisphere during this time. Although the diversity of the genera and species of the fauna is very low, the age and similarity of genera to those in Congo, west Africa, Arabia, Asia, Europe, and North, Central, and South America suggests that these genera were broadly distributed and diverse within the shallow marine settings of the Tethyan and southern provinces during middle and late Eocene.

## Introduction

Biogeographic origins of Madagascar’s modern biotic groups has been the focus of considerable research [1–4], yet the virtual absence of a Paleogene and Neogene terrestrial fossil record has left the origins of many of Madagascar’s living groups a mystery [2, 5]. Interpretations have been drawn largely from the negative evidence provided by a growing Late Cretaceous fossil record [5] and from molecular systematic analyses and the resulting deep estimated divergence dates [6–8]. However, a corresponding body of research is largely lacking for Malagasy marine organisms, despite the fact that nearly all of the known Cenozoic rocks are marine [9, 10].

Recent investigation in marine carbonate deposits exposed in the Mahajanga Basin has yielded Eocene marine vertebrates filling a gap in the Paleogene fossil record, including a primitive dugongid species belonging to *Eotheroides lambondrano* [11]. Here we report the first fossil neoselachian assemblage from the Eocene of Madagascar consisting of isolated teeth of eleven taxonomic groups, including first records of *Nebrius*, *Sphyrna* and *Pristis*. We include comparisons with other contemporaneous faunas and discuss the biogeographic implications and faunal similarities to other Eocene assemblages from Indian and East African Tethyan regions.

Currently, Madagascar’s neoselachian fossil record is represented by taxa from the Late Cretaceous [12] and Miocene [13, 14]; other associated marine taxa including foraminifers, corals, echinoderms, sponges, gastropods, bony fish, sea cows, turtles and crocodilians [11, 13, 15–17]. Cretaceous neoselachians include both batoid remains (*Parapalaeobates*: Rhinobatidae and *Brachyrhizodus*: Mylobatidae), and sharks (*Carcharias*, two species of *Squalicorax* and *Cretalamna*, and a single species of *Serratolamna*) from the Maastrichtian of northwestern Madagascar [12]. Miocene neoselachians include the sharks *Otodus megalodon*, *Carcharias*, *Galeocerdo*, *Rhizoprionodon*, *Sphyrna*, *Hemipristis*, and rays *Squatina*, *Rostroraja*, *Himantura*, *Myliobatis* [14]. Teleost fish have also been recovered, predominantly barracuda (*Sphyraena* sp.) [15].

### Geology

Tertiary sedimentary rocks in the Mahajanga basin are exposed along a NE-SW arch parallel to the shoreline of the Mozambique Canal. These rocks are exposed for few hundred kilometers, and overlay thick Cretaceous siliciclastic, carbonate, and volcanic units [9, 18].

**Fig 1:**
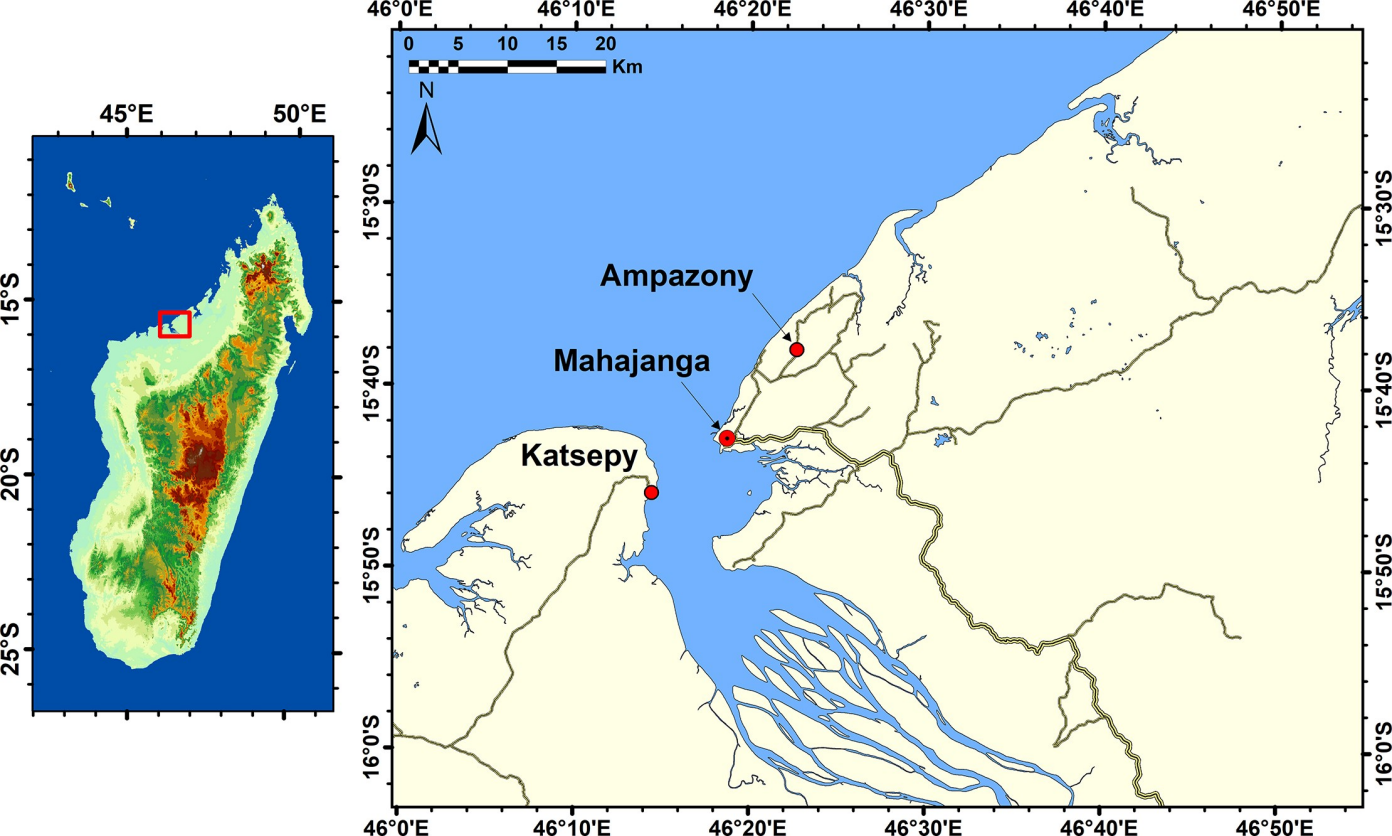
Map showing location of study localities: Ampazony and Katsepy, northwestern Madagascar. Also indicated is the port city of Mahajanga. Elevation model from Aster Global Digital Elevation Model (https://asterweb.jpl.nasa.gov/gdem.asp).

Fossils were surface collected from two regions: 1) Ampazony, approximately 15 km northeast of Mahajanga, and 2) Katsepy, west of Mahajanga across the Betsiboka River (Fig 1). Sediments at Ampazony consist of interbedded sandy claystones, mudstones, siltstones, and marly limestones that accumulated in low-lying coastal and shallow marine environments. Fossiliferous beds also produced invertebrate fossils, including bivalves, and gastropods, fragmentary remains of bony fishes and reptiles (fragments of turtle carapace and plastron and crocodyliform teeth), and the nearly-complete skull of a sirenian mammal [11]. Nearby underlying exposures display large-scale mudcracks that are consistent with subaerial exposure, presumably on peritidal mudflats. Fossil-bearing rocks were previously mapped as Pliocene [18] but have been reinterpreted as middle to late Eocene (see [11] and below).

Sediments at Katsepy are exposed on the shore face of the Mozambique Canal, as cliffs and large overturned blocks of carbonate units. These consist largely of nummulitic limestones with numerous other foraminiferans (mostly alveolinids), crinoids, echinoids, bivalves, gastropods, crabs, and occasionally vertebrate fossils (bony fishes, sharks, and rays). These nearshore marine sediments have been described as Eocene in age [9].

## Materials and methods

Isolated teeth, dental plates, and stingray spines comprise the majority of elements obtained in this study. Fossils were collected by surface prospecting and collecting over outwashes and dry sediment sieving through 2 mm and 5 mm sieves. Most of the isolated teeth exhibit very good preservation of both crown and root and show no signs of reworking or transportation. Some teeth are broken, but this appears to be the result of recent weathering and sediment washing. All teeth and dental material were cleaned with distilled water using soft brushes to remove salt film and sediment residue and were dried at normal room temperature. A thin acetone-based adhesive was applied around weakness areas and cracks. Teeth originally preserved in carbonate rocks were removed mechanically by using # 4 and # 3 (thin-headed) airscribes. Measurements of complete and well-preserved elements were obtained in mm (height, width, and length) by using Mitutoyo digital calipers. Photographs and images of the teeth were acquired using a Nikon D7200 digital camera and scale bar and a 5MP Dino-Lite AM7915MZTL digital microscope. Systematics, distribution, and occurrences of fossil and Recent comparative material reported here are from Cappetta [19].

Specimen numbers are listed in Appendix 1. Tooth terminology follows Cappetta [19]. Tooth positions of lamniform taxa are "presumed" as detailed in Siversson et al [20]. The referral of any tooth morphotype to a particular file is speculative and may be open to alternative interpretation.

**Nomenclatural Acts**—The electronic edition of this article conforms to the requirements of the amended International Code of Zoological Nomenclature, and hence the new names contained herein are available under that Code from the electronic edition of this article. This published work and the nomenclatural acts it contains have been registered in ZooBank, the online registration system for the ICZN. The ZooBank LSIDs (Life Science Identifiers) can be resolved and the associated information viewed through any standard web browser by appending the LSID to the prefix “http://zoobank.org/”. The LSID for this publication is: urn:lsid:zoobank.org:pub: urn:lsid:zoobank.org:pub:BE4A8582-F187-454F-949F-C5612EC9F577. The electronic edition of this work was published in a journal with an ISSN, and has been archived and is available from the following digital repositories: PubMed Central, LOCKSS.

**Institutional Abbreviations**—**UAP**, Université d’Antananarivo, Antananarivo, Madagascar. Illustrated specimens are indicated in bold typeface.

### Systematic Paleontology

                            Class CHONDRICHTHYES Huxley, 1880

                            Order ORECTOLOBIFORMES Applegate, 1972

                            Family GINGLYMOSTOMATIDAE Gill, 1862

                            Genus *Nebrius* Rüppell, 1837

                            *Nebrius blankenhorni* Stromer, 1905

                            Fig 2A–2C.

**Fig 2:**
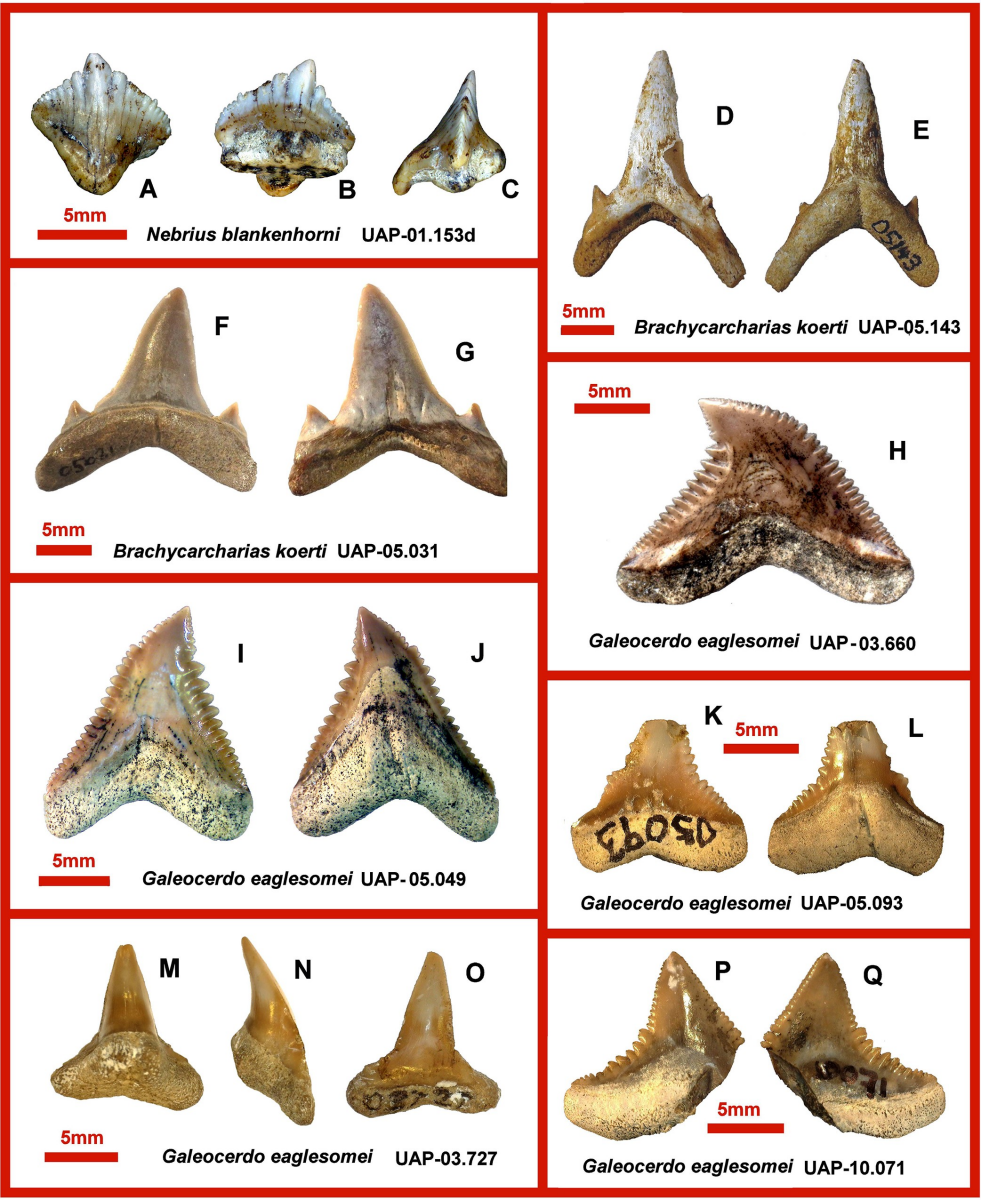
Eocene shark teeth from northwestern Madagascar. **A, B, C.** UAP-01.153d *Nebrius blankenhorni*, anterolateral tooth in labial (A), basal (B), and lateral views. **D, E.** UAP-05.031 *Brachycarcharias koerti*, left upper anterior tooth in labial (E) and lingual views (F). **F, G.** UAP-05.031 *Brachycarcharias koerti*, lower lateral tooth in lingual (F) and labial views (G). **H.** UAP-03.660 *Galeocerdo eaglesomei*, upper anterolateral tooth in labial view. **I, J.** UAP-05.049 *Galeocerdo eaglesomei*, upper anterior tooth in labial (I) and lingual views (J). **K, L.** UAP-05.093 *Galeocerdo eaglesomei*, lower parasymphyseal tooth in labial (K) and lingual views (L). **M, N, O.** UAP-03.727 *Galeocerdo eaglesomei*, lower parasymphyseal tooth in lingual (M), lateral (N) and labial views (O). **P, Q.** UAP-10.071 *Galeocerdo eaglesomei*, upper parasymphyseal tooth in lingual (P) and labial views (Q).

Material: One anterolateral tooth (see Appendix 1 for the specimen numbers of these and subsequent species).

### Description

UAP-01.153d is 5.5 mm in total height, 7.9 mm in mesiodistal width, and is 6.0 mm in labiolingual thickness. The crown compromises most of the tooth. It is broad labially, asymmetrical labiolingually and mediolaterally with strongly serrated cutting edges on both mesial and distal of the apex of the crown. The distal cutting edge is concave, the mesial slightly convex. A large labial apron (almost a tongue) tapers to overhang base of root. The lingual face possesses a less pronounced protuberance, ending at base of root. The root is broad laterally but thin vertically; the basal face is broad and flat, and possesses a central foramen.

### Discussion

*Nebrius blankenhorni* has been previously recorded from the mid to late Eocene of Egypt [21, 22]. It is particularly common in the late Lutetian of the Midawara Formation in the Fayum region, Western Desert of Egypt [23] and the middle Eocene of Togo [24]. It typically has large teeth which usually have a series of fine apically directed cracks in the enameloid on the labial crown.

                            Order LAMNIFORMES Berg, 1958

                            Family ODONTASPIDIDAE Müller& Henle, 1839

                            Genus *Brachycarcharias* Cappetta & Nolf, 2005

                            *Brachycarcharias koerti* Stromer, 1910

                            Fig 2D–2G.

Material: three teeth.

### Description

The left upper anterior tooth UAP-05.143 (Fig 2D and 2E) is 21.8 mm in total height, 16.4 mm in mediolateral width, and 6.4 mm in anteroposterior thickness. The tooth crown is tall and gracile but corroded by ongoing weathering. The crown is narrow and slim, the root lobes form a “V” shape. The lateral cusps are small, conical, multiple on the mesial root. There is a pronounced nutrient groove. Its relative size and narrow crown suggest that it is a tooth from a juvenile individual.

The left upper lateral tooth UAP-05.031 (Fig 2F and 2G) is 16.9 mm in total height, 17.4 mm in mediolateral width, and 6.1 mm in anteroposterior thickness. The tooth crown is large, basally wide and robust. The lateral cusps large triangular, spatulate and slightly laterally directed. The root lobes are widely separated forming a distinctive “V” shape in labial view, lingual apron of the root pronounced nutrient groove.

### Discussion

Previously the species *koerti* has been referred to the genus *Otodus* [25] *Carcharias* [26], *Lamna* [27], *Odontaspis* [28], *Cretalamna* [29] and *Serratolamna* [30]. In this paper, following Cappetta et al [31] and Underwood et al [23], *koerti* is referred to the genus *Brachycarcharias* because of its close similarity to the type species *B*. *lerichei* [32]. Both resemble *Carcharias* as juveniles and develop less needle-like and more triangular crowns with age, both in anterior and in the lateral teeth.

*Brachycarcharias koerti* is well known from Africa [24, 25, 28, 33–36] and middle Eocene sediments of North and South Carolina, USA [37–39]. Stromer [25] in his type description only figured two teeth from the middle Eocene phosphates of Togo. However, a series of teeth from Lutetian of Ameki, southern Nigeria were figured by White [28] which form the basis of our concept of the species. More recently large numbers of *B*. *koerti* have become commercially available from the type locality in Togo, which confirm White’s determination. Cappetta & Case [40] described *Tethylamna dunni*, a new genus and species of odontaspid from the mid-Lutetian, (middle Eocene) Lisbon Formation of Andalusia, Alabama, USA. The figured teeth closely resemble those of *B*. *koerti* and may be conspecific. Specimens of *B*. *koerti* from the Comfort member of the Castle Hayne Limestone in North Carolina tend to be larger with wider flatter crowns than those from Togo [38]; (DJW pers. obs).

*B*. *koerti* can be distinguished from the superficially similar species *Tethylamna twiggsensis* [27] by the shape and direction of the lateral cusps, which in the latter are more triangular, laterally recurved and often multiple [40].

A tooth figured by Casier (fig 5 in [41]) from the middle Eocene Midra Shale of Qatar (as “*Lamna” gafsana*) is probably from *B*. *koerti* and bears a close resemblance to UAP-05-031.

                            Order CARCHARHINIFORMES Compagno, 1973

                            Family CARCHARHINIDAE Jordan and Evermann, 1896

                            Genus *Galeocerdo* Müller & Henle, 1837

**Fig 3:**
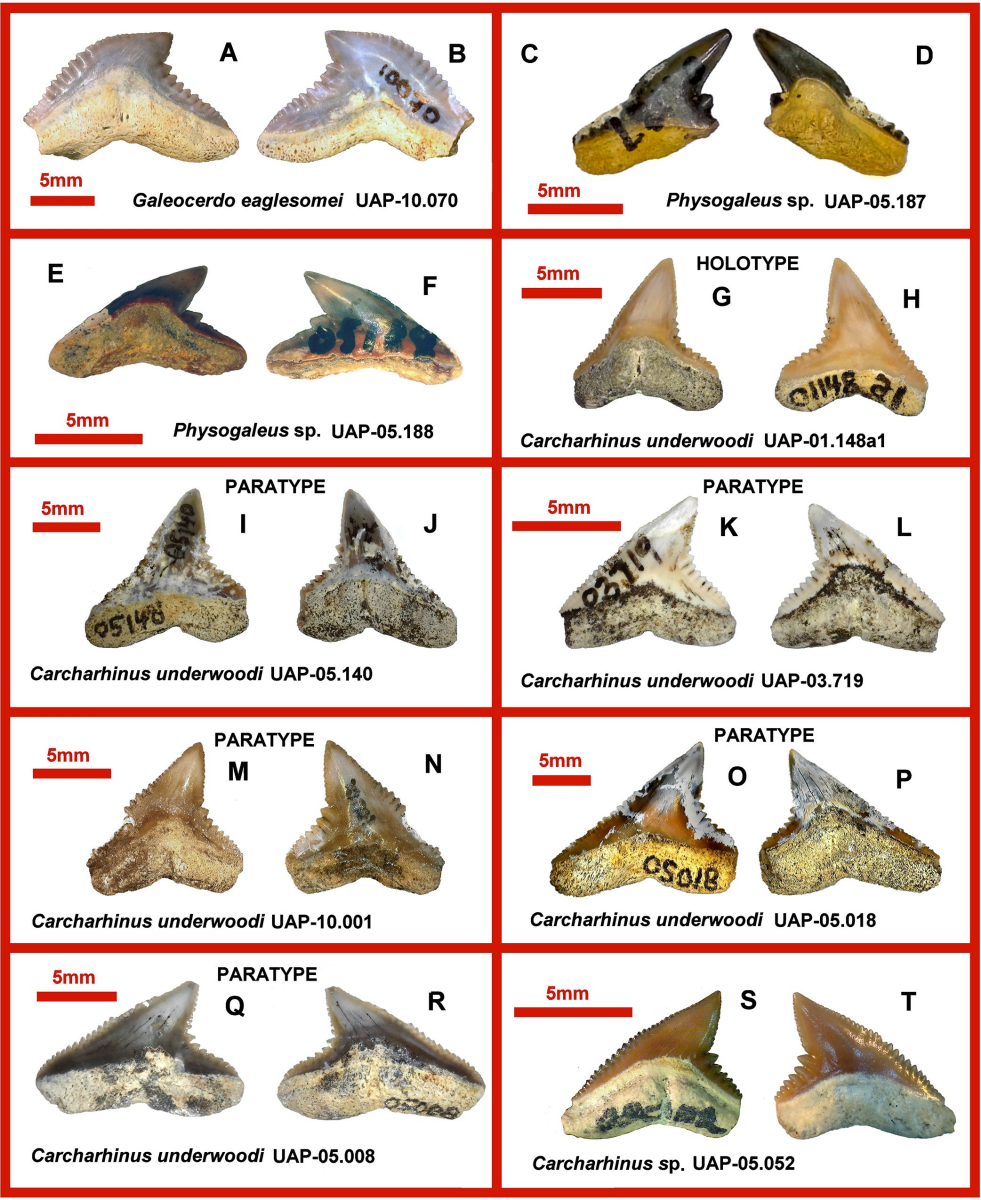
Eocene shark teeth from northwestern Madagascar. **A, B.** UAP-10.070 *Galeocerdo eaglesomei*, upper lateral tooth in lingual (A) and labial views (B). **C, D.** UAP-05.187 *Physogaleus* sp., lower anterolateral tooth in labial (C) and lingual views (D). **E, F.** UAP-05.188 *Physogaleus* sp., upper anterolateral tooth in lingual (E) and labial views (F). **G, H.** UAP-01.148a1 *Carcharhinus underwoodi*, HOLOTYPE, upper anterior tooth in lingual (G) and labial views (H). **I, J.** UAP-05.140 *Carcharhinus underwoodi*, PARATYPE, upper anterolateral tooth in labial (I) and lingual views (J). **K, L.** UAP-03.719 *Carcharhinus underwoodi*, PARATYPE, upper anterolateral tooth in labial (K) and lingual views (L). **M, N.** UAP-10.001 *Carcharhinus underwoodi*, PARATYPE, upper anterolateral tooth in lingual (M) and labial views (N). **O, P.** UAP-05.018 *Carcharhinus underwoodi*, PARATYPE, upper anterolateral tooth in labial (O) and lingual views (P). **Q, R.** UAP-05.008 *Carcharhinus underwoodi*, PARATYPE, upper lateral tooth in labial (Q) and lingual views (R). **S, T.** UAP-05.052 *Carcharhinus* sp., tooth in lingual (S) and labial views (T).

                            *Galeocerdo eaglesomei* White, 1955

                            (Fig 2H–2Q, Fig 3A–3D)

Material: 31 isolated teeth.

### Description

The teeth exhibit broad, triangular blades and serrations on the mesial and distal cutting edges with a distally-bent cusp. The mesial heel is concave, the distal heel is convex. The distal heel is longer than the mesial, possessing finer serrations and a shorter cutting edge. The serrations are simple, never compound. The root has higher lingual face; the labial surface is concave. A strong nutritive groove bisects the lingual aspect of the root.

UAP-03.660 (Fig 2H), is 20.0 mm wide and 14.2 mm high. It has a wide triangular crown that extends laterally over the root lobes. The mesial cutting edge is coarsening midway up the crown becoming more finely serrated towards the apicodistally directed tip. The distal cutting edge coarsens apically as far as the notch, above which it is more finely and less regularly serrated. The labial surface is essentially planar with a slight medially placed depression at the base and a slightly inflated apex. The basal ledge is shallow and has a thin covering of enameloid. The root occupies the bulk of the lingual surface of the tooth. The enameloid covers just the tip and extends as a strip laterally on either side of the lingual protuberance which bears a medially situated indistinct, apicobasally directed, nutritive groove.

UAP-05.049 (Fig 2I and 2J), is 14.9 mm wide and 17.1 mm high. This tooth is essentially similar in general design to that of UAP-03.660 but is relatively taller and narrower, with a more apically directed crown tip and more closely spaced root lobes.

UAP-05.093 (Fig 2K and 2L), is 11.5 mm wide (height cannot be reliably measured). This tooth has a triangular crown narrowing apically, a low robust, inflated, root, a relatively large lingual protuberance and distinct nutritive groove. The distal cutting edge is concave and coarsely serrated, the (damaged) mesial cutting edge is less coarsely serrated.

UAP-03.727 (Fig 2M–2O), is 11.7 mm wide and 14.9 mm high and 4.6 mm labiolingually deep. This tooth has a narrow upright crown with narrow shoulders. The labial face is lightly convex, the lingual face is strongly convex. The cutting edge is faintly serrated. The root is compact, robust and rounded lacking an obvious nutritive groove.

UAP-10.071 (Fig 2P and 2Q), is broken (width cannot be reliably measured) and is 8.6 mm high. This tooth has a low triangular crown with a relatively large apically directed tip. The root is damaged but appears to be similar but wider to that of UAP-05.093.

UAP-10.070 (Fig 3A and 3B), is broken (width cannot be reliably measured) and 12.9 mm high. This is a wide, low-crowned tooth with a distally directed finely serrated crown tip. The labial crown face is relatively flat with a moderately large basal ledge. The root is lingually low with a large nutritive groove bearing two small vascular foraminae.

### Discussion

White [28] described a number of isolated *Galeocerdo* teeth from the Eocene of Ameki, Nigeria which he initially identified as *G*. *latidens* Agassiz, 1843. The age of White’s Ameki material has been questioned by Underwood & Gunter [42] who suggest that it could be late Eocene.

Using *G*. *cuvier* as a model, White suggested that the teeth with more distally directed crown tips were from the lower jaw. He later recognized [43] that the degree of heterodonty they exhibited was far greater than seen in the Recent species *Galeocerdo cuvier* Péron & Lesueur, 1822, which displays virtually no dignathic and minimal monognathic heterodonty [44]. He described these teeth as a new species, *Galeocerdo eaglesomei*. Although *G*. *eaglesomei* is a relatively well-known tooth-based species, teeth other than the large upper anteriolaterals have been rarely figured.

Based on the dental reconstructions of *Physogaleus* spp. [45, 46] UAP-05.093 would be a lower anterior and UAP-3.727, a lower parasymphyseal. UAP-10.071 would be an upper parasymphyseal. UAP-05049 is an upper anterior tooth and UAP-03.660 an upper anterolateral tooth. UAP-10.070 is probably an upper lateral tooth.

*G*. *eaglesomei* has been recorded from the north Africa [24, 25, 26, 35, 37], the Middle East [39] and the USA [47, 48]. Adnet et al [36] noted that teeth of *Galeocerdo* from the late Eocene of Dakhla, south-west Morocco were almost double the size of White’s types from the late Eocene of Ameki, Nigeria and those from the middle Eocene of Togo and that an increase in size in the younger members of the “lineage” was conceivable. The Ampazony specimens are consistent in size with White’s types. This does not necessarily indicate that they are coeval, the Ampazony teeth could be from an ontogenetically younger population.

Adnet et al [49] described some new carcharhinid sharks from the late Eocene and early Oligocene of Pakistan, including a new species of *Carcharhinus*, *C*. *balochensis*. The figured specimens with one exception can be referred to *Galeocerdo eaglesomei*. The holotype, (Fig 3, 5–6 in [49]) an incomplete upper right tooth, closely resembles a tooth of *G*. *eaglesomei* figured from the middle Eocene Castle Hayne Limestone of North Carolina by Case and Borodin [48]. One of the teeth figured by Adnet et al (Fig 3, 7–9 in [49]) from the early Oligocene displays complex serrations, a character not present in middle Eocene populations of *G*. *eaglesomei* but present in the similar Miocene species *G*. *mayumbensis* [14]. This specimen, assuming that the dating is correct, extends the range of *G*. *eaglesomei* into the early Oligocene. One of the remaining figured specimens (Fig 3, 10–11 in [49]) can be referred to *Carcharhinus* and is discussed further below.

The name *Galeocerdo eaglesomei* is used here to accommodate the Nigerian type series which are relatively small [28], the very similar teeth albeit much larger from the Bartonian of the Fayum [23] and Priabonian of south-west Morocco [37] and a large specimen with complex serrations from the early Oligocene of Pakistan [48]. Both Adnet et al [36] and Underwood et al [23] have suggested that they might represent different species. More well-dated material of this species, or species-group is needed to resolve this. The Madagascan specimens do not help clarify their age or taxonomic relationships.

Similarly, the species *G*. *latidens*, *G*.*eaglesomei* and *G*. *mayumbensis* appear to be members of a separate lineage occupying warm equatorial waters between middle Eocene and late Miocene times. Along with the Eocene species *Galeocerdo latidens* Agassiz 1843 the teeth differ from those of *G*. *cuvier* in their degree of dignathic heterodonty and partial loss of the notch in the distal cutting edge. These differences suggest that they should be referred to a separate new genus.

                            Genus *Physogaleus* Cappetta, 1980

                            *Physogaleus* sp.

                            (Fig 3C–3F)

Material: Two teeth.

### Description

UAP-05.187, (Fig 3C and 3D) is a left lower anterolateral tooth lacking its distal root lobe. Its (incomplete) mediolateral width is 9.3 mm and height is 10.1 mm. The tooth has a tall apicodistally directed crown and at least one cusplet on the distal shoulder. The mesial shoulder is damaged but shows signs of five or six small serrations. Little of the root is exposed in labial view. Lingually the tooth has a large, centrally placed protuberance divided by a shallow apically directed groove containing a nutrient foramen.

UAP-05.188, (Fig 3E and 3F) is 5.1 mm in total height, 9.5 mm in mediolateral width. The tooth has an apicodistally directed crown and two distinct cusplets on the distal shoulder. Little root is exposed on the labial view. Lingually the tooth has a large, centrally placed protuberance; however, its poor state of preservation obscures the vascularisation. Two small centrally placed foraminae occur just basal to the root/crown junction. The root occupies the basal 25% of the labial surface. The mesial cutting edge is straight and coarsely serrated at the base.

### Discussion

Tooth UAP-05.187 is consistent in morphology with lower teeth of the highly heterodont species or species group commonly referred to as *P*. *secundus*. Specimen UAP-05.187 has a more generalized carcharhinid tooth morphology, consistent with that of *Physogaleus*, but equally with other small carcharinid sharks like *Eogaleus*, *Abdounia*, *Paragaleus*. Similar teeth are abundant in the middle Eocene of the Fayum, Egypt (DJW pers. obs) and can be separated from those of *Rhizoprionodon* and *Sphyrna* by the presence of multiple distal cusplets.

                            Genus *Carcharhinus* Blainville, 1816

                            *Carcharhinus underwoodi* sp. nov. urn:lsid:zoobank.org:act:E8429AD0-B8C9-4E01-ADE5-E4B0DBF15152

                            Fig 3G–3R

                            Synonymy:

? 1990 *Carcharhinus* sp. 1. Case & Cappetta [50], Pl. 7, figs 164–165.

? 2008 *Carcharhinus balochensis* Adnet et al [49], Fig 3, 10–11 (not 1–9 & 12–19).

Material: 12 isolated teeth including the figured holotype (UAP-01.148a1) and five paratypes.

Etymology: Named in honor of Dr. Charlie Underwood in recognition of his work on fossil sharks and rays in general and carcharhiniform sharks in particular.

**Type Locality:** Ampazony, approximately 15 km northeast of Mahajanga, Madagascar.

**Type stratum:** Unnamed marine horizon overlying an omission surface with desiccation cracks.

**Age:** middle-late Eocene [11].

**Diagnosis:** *Carcharhinus* species known only from isolated teeth. The teeth display gradient monognatic heterodonty, but no evidence of dignathic heterodonty. The teeth are slightly wider than tall and comprise a distally directed crown and distal heel. The mesial cutting edge is straight to slightly convex. The distal serrae are triangular and evenly increase in size from the root-crown junction to the rounded distal notch. The mesial and distal cutting edges on the crown apex are finely serrated, the basal portion of the mesial cutting edge is irregularly serrated. The angle subtended by the crown apex is about 40° for anterior teeth increasing to 50° in more lateral files.

### Description

**Fig 4:**
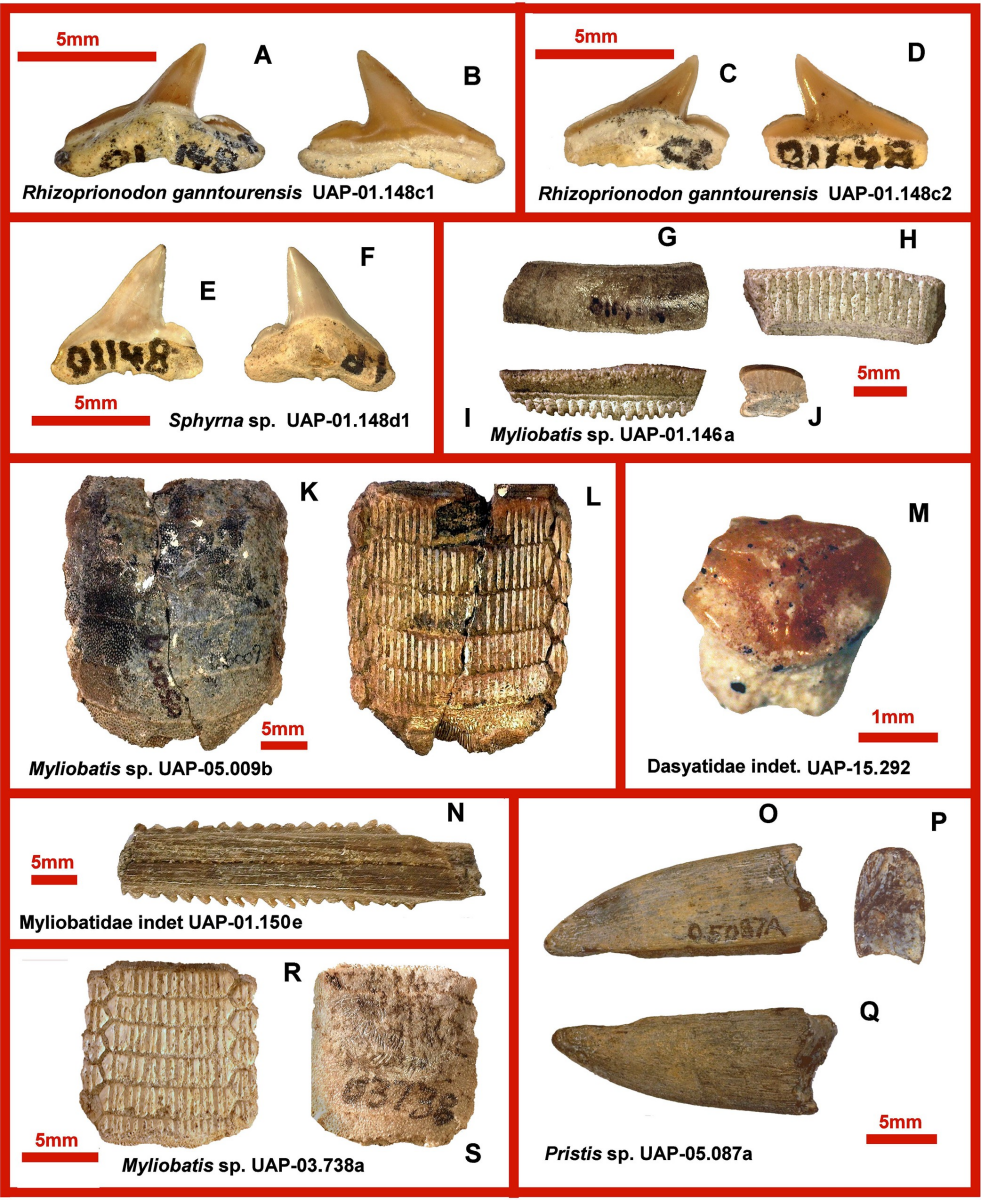
Eocene shark and ray remains from northwestern Madagascar. **A, B.** UAP-01.148c1 *Rhizoprionodon ganntourensis*, tooth in lingual (A) and labial views (B). **C, D.** UAP-01.148c2 *Rhizoprionodon ganntourensis*, tooth in lingual (C) and labial views (D). **E, F.** UAP-01.148d1 *Sphyrna* sp., tooth in labial (E) and lingual views (F). **G, H, I, J.** UAP-01.146a *Myliobatis* sp., tooth in occlusal (G) basal (H), and lateral views (I), and in section (J). **K, L.** UAP-05.009b *Myliobatis* sp., partial upper dentition in occlusal (K) and basal views (L). **M.** UAP-15.292 Dasyatidae indet., tooth in lingual view. **N.** UAP-01.150e Myliobatidae indet., tail spine. **O, P, Q.** UAP-05.087a *Pristis* sp., rostral spine in lateral (O, P), and basal views (Q). **R, S.** UAP-03.738a *Myliobatis* sp., partial lower dentition in basal (R) and occlusal views (S).

The holotype UAP-01.148a1; (Fig 4G and 4H) is 9.5 mm wide and 10mm high. The crown is roughly triangular with a slightly distally directed cusp. The labial crown surface is flat to slightly convex with no obvious basal ledge. There are serrae (5/mm) becoming more finely serrate towards the tip (6/mm). The distal cutting edge of the cusp is slightly convex and lightly serrated (6/mm). At the base of the cusp the edge curves abruptly through an angle of 50° to form a rounded notch. The distal base of the crown bears ten serrae (2/mm) becoming smaller towards the root/crown junction. On the lingual surface the root occupies the basal 45% of the tooth with a prominent protuberance divided by a distinct nutritive groove and a slit-like centrally placed foramen.

Paratype: UAP-05.140 (Fig 3I and 3J). This tooth is 12.3 mm wide and 12.3 mm high and thus slightly larger than the holotype but similar in most respects. The mesial cusp is slightly more convex towards its tip and the mesial root lobe is longer. There are eight serrae on the distal cutting edge reaching just apical to the curved notch.

Paratype: UAP-03.719 (Fig 3K and 3L). This tooth is 7.9 mm wide and 6.6 mm high. It is slightly smaller than the holotype and has a smaller, more distally directed cusp. The distal notch is slightly more angular. The lingual protuberance is less prominent.

Paratype: UAP-10.001 (Fig 3M and 3N). This tooth is 10.3 mm wide and 9.7 mm high. The cusp is smaller and the space occupied by the root on the lingual surface is larger than that of the holotype.

Paratype: UAP-05.018 (Fig 3O and 3P). This tooth is 16.5 mm wide and 13.9 mm high. The entire mesial cutting edge and that of the distal cusp are virtually straight giving the tooth a more angular aspect. On the lingual face, the protuberance on is less pronounced and the root extends more than half way up the tooth.

Paratype: UAP-05.008 (Fig 3Q and 3R). This tooth is 13.1 mm wide and 8.4 mm high. The crown is relatively low and wide with a distally directed small cusp. Unlike the previous teeth which have a notch of between 130° and 135°, this tooth measures about 110°.

In addition to the specimens from Madagascar, there are two possible records of *Carcharhinus* attributable to *C*. *underwoodi* in the literature. Case and Cappetta [50] figured a tooth as from the late middle Eocene of the Fayum, Egypt as “*Carcharhinus* sp. 1. It is very similar those of *C*. *underwoodi* in size, shape, and degree of serration. Similarly, one of the teeth from the late Eocene of Pakistan, figured by Adnet et al (Fig 3, 10–11 in [49] as *Carcharhinus balochensis* closely corresponds to those of *C*. *underwoodi*.

### Discussion

*Carcharhinus* has a global distribution from the late Eocene to the Recent and is represented by 35 Recent species [51, 52]. The majority of these exhibit strong dignathic heterodonty; upper teeth are generally triangular, serrated and distally inclined whereas lower teeth bear narrow lightly serrated, apically directed crowns. No carcharhinid teeth with wide crown bases and narrow upright crowns, typical of lower teeth of *Carcharhinus*, *Negapriodon and Aprionodon*, were encountered at Ampazony, which suggests that teeth of *C*. *underwoodi* did not exhibit strong dignathic heterodonty. There are just two Recent species of *Carcharhinus* that lack strong dignathic heterodonty; *C*. *ambionensis* and *C*. *leucas*. Teeth of *C*. *underwoodi* are less distally inclined and having a straighter mesial cutting edge than those of *C*. *ambionensis* and *C*. *leucas*. Additionally, anterior teeth of *C*. *ambionensis* are upright and almost symmetrical, a character not seen in the limited material of *C*. *underwoodi*. Teeth of *C*. *leucas* are generally wider than those of *C*. *underwood* with an apical angle exceeding 50° [52]. An unnamed species of *Carcharhinus*, with teeth very similar to those of *C*. *leucas* occurs in the late Eocene of south-western Morocco (Fig 3G in [36]) and the Fayum in Egypt (Fig 3N in [23]). These can separated from teeth of *C*. *underwoodi* in having a much wider triangular tooth with strong distal notch.

Middle Eocene records of *Carcharhinus* ssp. are few and are reviewed by Underwood and Gunter [42]. They figure a single unnamed species from the middle/late Eocene of Jamaica, similar to the specimens described herein but differing in having finer and less regular mesial serrations and a sharper distal notch. This unnamed species is probably the oldest figured example of *Carcharhinus*. The remaining middle Eocene species referred to *Carcharhinus* are better accommodated in other genera including *Negaprion* and *Aprionodon* [19, 53]. The upper tooth of *Carcharhinus* figured by Kemp et al (Pl. 7 Fig 8 in [54]) from the middle Eocene of southern England, is a misidentification of an upper tooth of an undescribed *Physogaleus*. The lower tooth (Pl. 7 Fig 9 in [54]) is *Negaprion marcaisi* Arambourg, 1952 ([35]; DJW unpublished data), previously unrecorded from the UK.

*Carcharhinus underwoodi* is the oldest named species of *Carcharhinus*. Its teeth are close in shape and have a similar degree of dignathic heterodonty as the Recent species *C*. *ambionensis*. The oldest named *Carcharhinus* species displaying a dignathic heterodonty and serration pattern typical of Recent species, like *C*. *perezii*, *C falciformis and C*. *leiodon* would appear to be *C*. *elongatus* Leriche, 1910 known from the early Oligocene of western Europe [45, 52].

                            Genus *Carcharhinus* Blainville, 1816

                            ? *Carcharhinus* sp.

                            Fig 3S and 3T

Material: One isolated tooth.

### Description

UAP-05.052 (Fig 3S and 3T) is 6.6 mm wide and 6.1mm high. The labial aspect of the crown is slightly convex with a distally directed cusp and virtually no basal ledge. The cutting edge is continuous and serrated. The mesial cutting edge is convex with serrae increasing in size towards its midpoint where there are 3 serrae/mm and disappearing on the slightly upturned tip of the cusp. The distal cutting edge of the cusp is also convex and faintly serrated (5/mm). At its base, there is a sharp angle where the distal crown slopes toward the tooth edge. The cutting edge of the shoulder bears seven serrae decreasing in size basally. The lingual root is low, with a small protuberance with a wide nutritive groove.

### Discussion

This tooth is reminiscent of both *Galeocerdo* and *Carcharhinus*. It differs from *C*. *underwoodi* in having evenly spaced serrae on the mesial cutting edge and a pronounced distal notch.

                            Genus *Rhizoprionodon* Whitley, 1929

                            *Rhizoprionodon ganntourensis* Arambourg, 1952

                            (Fig 4A–4D)

Material: 7 isolated teeth.

### Description

UAP-01.148c1 (Fig 4A and 4B), is 7.5 mm wide and 6.6 mm high. This tooth has a distally directed cusp, a complete cutting edge and distal heel. The root has a rectilinear basal margin and distinct nutrient groove.

UAP-01.148c2 (Fig 4C and 4D), is 7.5 mm wide and 6.6 mm high. This tooth is similar to the previous tooth, differing in having a slightly apically recurved cusp.

### Discussion

Teeth of *Rhizoprionodon* are present in most middle Eocene deposits worldwide and do not differ significantly from their Recent counterparts. They exhibit gynandric heterodonty, lower teeth of adult males having somewhat sigmoid apically curved crowns.

                            Family Sphynidae Gill, 1872

                            Genus *Sphyrna* Rafinesque, 1810

                            ? *Sphyrna* sp.

                            (Fig 4E and 4F)

Material: Two teeth.

### Description

UAP-01.148d1 (Fig 4E and 4F), is 6.85 mm wide and 5.9 mm high. This tooth has a distally directed cusp, a complete cutting edge and distal heel. The shoulder is rounded and smooth. The root possesses a rectilinear basal margin and distinct nutrient groove.

### Discussion

Teeth of this species are similar in design to those of *Rhizoprionodon* or *Scoliodon*, but much higher crowned. More material is needed to confirm this record. They are similar to an unnamed specimen figured by Underwood et al (Pl. 5 Fig S in [23]). Adnet et al [36] recorded *Sphyrna* sp. from the middle-late Eocene in southwestern Morocco.

                            Superorder BATOMORPHII Cappetta, 1980

                            (= BATOIDEA Compagno, 1973)

                            Order MYLIOBATIFORMES *sensu* Compagno, 1973

                            Family MYLIOBATIDAE Bonaparte, 1838

                            Genus *Myliobatis* Cuvier, 1817

                            *Myliobatis* sp.

                            (Fig 4G–4L, 4R and 4S)

Material: 632 individual teeth and tooth plates.

### Description

By far the most common vertebrate remains from the Ampazony locality are fragments and occasional palates of *Myliobatis*. For the moment, because of their fragmentary nature, they remain in open nomenclature.

UAP-01.146 a (Fig 4G–4J) is an incomplete chevron. The rectilinear shape and the slight bulge on the occlusal surface suggests that it is from the lower dentition.

UAP-05.009b: (Fig 4K and 4L) This is a fairly robust partial upper palate showing a median row and the first lateral row on either side. It resembles both *M*. *dixoni* Agassiz 1843 and *M*. *striatus* Buckland 1837.

UAP-03.738a: (Fig 4R and 4S) This is a small undetermined lower palate demonstrating predation damage on the occlusal surface.

### Discussion

Fragments of *Myliobatis* chevrons vastly outnumber any other shark or ray remains. The lack of diversity in myliobatiforms is unexpected. In similar assemblages elsewhere, it is not unusual to encounter a proportion of *Aetobatis*, *Rhinoptera*, *Leidybatis* and *Burnhamia*.

                            Myliobatiformes indet.

                            (Fig 4N)

Material: Four tail spines.

### Description

Tail spine (UAP-01.150e) is 32.4 mm in length, 7.8 mm in width, and 4.0 mm in thickness.

### Discussion

Tail spines are generally attributed to *Myliobatis*. However, the presence of other myliobatiforms necessitates a more open nomenclature.

                            Family DASYATIDAE Jordan, 1888

                            Dasyatidae indet.

                            (Fig 4M)

Material: One tooth.

### Description

The tooth (UAP-15.292) is 2.2 mm wide. It is too corroded to warrant a detailed description. There is a labial visor and a distinct medial lingual ridge flanked laterolingually by marginal hollows. The labial face is tabulate and lightly ornamented. Remains of a lingually displaced bilobed root are present.

### Discussion

This rather generalized morphology is characteristic of a number of genera including *Dasyatis*, *Himantura* and *Taeniura*.

                            Order RAJIFORMES Berg, 1940

                            Family PRISTIDAE Bonaparte, 1838

                            Genus *Pristis* Linck, 1790

                            *Pristis* sp.

                            (Fig 4O–4Q)

Material: 19 rostral spines.

### Description

Only UAP-05087a is well preserved, and is 22.8 mm in length, 8.8 mm in anteroposterior thickness and 5.3 mm in mediolateral dimensions. The spines are relatively long, with narrow anterior tip and pronounced groove on the posterior edge. Growth bands are visible in lateral and cross section.

### Discussion

Individual *Pristis* rostral pegs are generally considered indeterminate. Despite this, many authors refer Eocene specimens to *Pristis lathami* Galeotti 1837. Here, we prefer to leave the specimens collected in open nomenclature.

## Discussion and Conclusions

### The age of the Ampazony and Katsepy deposits

To date the Ampazony sediments have yielded no biostratigraphically useful microfossils, although further micropaleontological analyses are in progress to more precisely constrain the age of the deposits. The most compelling evidence for a middle to late Eocene age for the deposit is the presence of a skull of dwarf species of the halitherinid sirenian, *Eotheroides lambondrano* Samonds et al 2009 [11]. This genus ranges from the middle to late Eocene (Lutetian, Bartonian and Priabonian) in NW Africa and the USA and is common in the Priabonian of the Fayum, Egypt (Table 2 in [55]). Evidence suggesting a middle Eocene age for Ampazony is present in the size of the teeth of *Galeocerdo eaglesomei* which more closely match White’s Bartonian type specimens in size than those from the Priabonian of west Africa, although tooth size can be the result of ontogeny or environmental factors. The presence of *Nebrius blankenhorni*, previously recorded from the mid- to late Eocene of Egypt [21–23] and the middle Eocene of Togo [24] supports a middle to late Eocene age for the Ampazony and Katsepy deposits. Apart from usually lacking these two species, post-Eocene shark faunas are usually dominated by small species of *Carcharhinus*, particularly in inshore coastal or brackish deposits [19, 42].

The presence of thick nummulitic limestones at Katsepy is strongly indicative of a middle to late Eocene age but are also not conclusive. Assuming both localities are of similar age, as indicated by their elasmobranch tooth faunas, their most likely age, based on the evidence we have, is middle to late Eocene.

Eocene neoselachian faunas are widespread globally [20] and particularly rich in Pakistan [49], Egypt [23, 50], Jordan [56], West Africa [28], India [57], Morocco [58, 59], Europe [60], Antarctica [61–63], and North America [26, 55–57, 64, 65]. In contrast, published accounts of Paleogene and Neogene marine and terrestrial vertebrates from Madagascar are few [14].

The overall neoselachian fauna appears to bear a striking resemblance to that of several other locations, foremost among them Togo, Nigeria, Morocco and Egypt. Moroccan Eocene deposits from the Western Sahara have produced *G*. *eaglesomei*, *N*. *blankenhorni*, *Carcharhinus* and *Rhizoprionodon*, and *Myliobatis*. Egypt’s Eocene sediments are likewise populated with *G*. *eaglesomei*, *N*. *blankenhorni* and species of *Carcharhinus*, *Pristis* and *Myliobatis*. Strikingly, the sandshark genus *Striatolamia*, which dominates the European and north African middle Eocene, is absent along with most bottom feeding sharks and rays [32, 35, 64, 66–68]. These appear to have been replaced by *Brachycarcharias*, *Galeocerdo* and small carcharhiniforms. A similar pattern is seen in the Moroccan western Sahara [41] and Egypt [20, 31]. It is clear that, owing to the paucity of the material and the difficulty in collecting it, we are only seeing part of the picture. Considering Madagascar’s isolation in the Southern Ocean in the Eocene, further collecting with an emphasis on material less than 2 mm in size is likely to produce interesting results.

## Appendix 1

Catalogue numbers of material studied. Numbers in **bold** typeface are figured.

*Nebrius blankenhorni* **UAP-01.153d** (Ampazony)

*Brachycarcharias koerti* **UAP-05.143**, **UAP-05.031**(Ampazony); UAP-05.233 (Katsepy)

*Galeocerdo eaglesomei* UAP-01.148a2, UAP-01.148b1-01.148b5, UAP-01.150a, **UAP-03.660,** UAP-03.670, UAP-03.712, UAP-03.724, UAP-03.726, **UAP-03.727**,UAP-03.743, UAP-03.780a, UAP-03.780b, UAP-05.028, UAP-05.038, UAP-05.048, **UAP-05.049**,UAP-05.088a, UAP-05.088b, **UAP-05.093**, UAP-10.055, UAP-10.061, **UAP-10.070**, **UAP-10.071**,UAP-10.084, UAP-05.110 (Ampazony); UAP-05.190, UAP-05-198 (Katsepy)

*Physogaleus* sp.**UAP-05.187**, **UAP-05.188** (Katsepy)

*Carcharhinus underwoodi* **UAP-01.148a1**, **UAP-03.719**, UAP-03.725, UAP-03.749, UAP-05.004, **UAP-05.008**, **UAP-05.018**, UAP-05.037, UAP-05.077, UAP-05.117, **UAP-05.140**, **UAP-10.001** (Ampazony)

*Carcharhinus* sp. A. **UAP-05.052** (Ampazony)

*Rhizoprionodon ganntourensis* **UAP-01.148c1**, **UAP-01.148c2**, UAP-01.148c3, UAP-03.628, UAP-03.668 (Ampazony); UAP-05.189, UAP-05.192 (Katsepy)

*Sphyrna* sp.UAP-01.144a, **UAP-01.148d1** (Ampazony)

*Myliobatis* sp. **UAP-01.146a**, UAP-01.146 (lot of 72), UAP-01.150d, UAP-01.151a (lot of 77), UAP-01.151b (lot of 59), UAP-03.647, UAP-03.648 (lot of 17), UAP-03.716 (lot of 4), UAP-03.731 (lot of 48), **UAP-03.738a**, UAP-03.740, UAP-03.768 (lot of 4), UAP-03.772 (lot of 6), **UAP-05.009b**, UAP-05.012, UAP-05.039, UAP-05.054 (lot of 6), UAP-05.062 (lot of 23), UAP-05.062a, UAP-05.074, UAP-05.097, UAP-05.112, UAP-10.021 (lot of 9), UAP-10.028 (lot of 19), UAP-10.032, UAP-10.038 (lot of 11), UAP-10.051 (lot of 23), UAP-10.045, UAP-10.058 (lot of 10), UAP-10.083, UAP-10.093 (lot of 102), UAP-11.016, UAP-11.026 (lot of 29), UAP-11.033 (lot of 6), UAP-11.036, UAP-10.038 (lot of 11), UAP-11.043, UAP-11.046 (lot of 2), UAP-11.047, UAP-11.058 (lot of 22), UAP-11.067 (lot of 8), UAP-11.077 (lot of 19), UAP-11.091 (lot of 8), UAP-11.093 (lot of 3) (Ampazony); UAP-05.261; UAP-05.300; UAP-05.356 (lot of 2),UAP-05.184 (lot of 7), UAP-05.194, UAP-05.195, UAP-05.196, UAP-05.232 (Katsepy)

Myliobatidiform indet. tail spines **UAP-01.150e**, UAP-10.063, UAP-11.084, UAP-10.085 (Ampazony)

Dasyatidae indet. **UAP-15.292** (Katsepy)

*Pristis* sp.UAP-03.746, UAP-05.003, UAP-05.042, UAP-05.045, UAP-05.082, UAP-05.085 (lot of 5), UAP-05.087 (lot of 5), **UAP-05.087a**, UAP-05.130, UAP-10.002, UAP-10.033 (Ampazony)
